# Factors Influencing Malnutrition Among Older Adult Residents in the Western Region of Saudi Arabia: Sex Differential Study

**DOI:** 10.2196/55572

**Published:** 2024-09-16

**Authors:** Mai Adil Ghabashi, Firas Sultan Azzeh

**Affiliations:** 1 Clinical Nutrition Department, Faculty of Applied Medical Sciences Umm Al-Qura University Makkah Saudi Arabia

**Keywords:** Elderly, Makkah, Malnutrition, Mini Nutritional Assessment Short Form, Risk factors

## Abstract

**Background:**

The global population of older adults is on the rise. As people age, their physical functions gradually decline, leading to a decrease in the overall functioning of different organ systems. Due to these changes, older individuals are at a higher risk of encountering various adverse health outcomes and complications, such as malnutrition.

**Objective:**

This study aims to investigate the prevalence of malnutrition and its associated factors among older adults dwelling in the western region of Saudi Arabia. We have analyzed these factors separately for both men and women to understand any potential sex differences.

**Methods:**

A nonrandomized cross-sectional study was conducted for older adults aged ≥60 years in the western region of Saudi Arabia. Personal information was obtained through a closed questionnaire. The Mini Nutritional Assessment Short Form was used to determine the malnutrition status of older adults. Consequently, the individuals were divided into 2 groups: normal and malnourished. To assess the risk factors related to malnutrition, the odds ratio (OR) and 95% CI were determined using a binary logistic regression.

**Results:**

The prevalence of malnutrition in men and women was around 7% and 5%, respectively. Potential risk factors related to malnutrition in men were higher age (OR 1.263, 95% CI 1.086-1.468; *P*=.002), being widowed (OR 8.392, 95% CI 1.002-70.258; *P*=.049), and having dental problems (OR 9.408, 95% CI 1.863-47.514; *P*=.007). On the other hand, risk factors associated with malnutrition in women were lower BMI (OR 0.843, 95% CI 0.747-0.952; *P*=.006) and being disabled (OR 18.089, 95% CI 0.747-0.952; *P*=.006).

**Conclusions:**

The findings of this study provide important insights into the risk factors for malnutrition among older adults in the western region of Saudi Arabia. While the overall prevalence of malnutrition was relatively low, the analysis revealed distinct risk factors for older men and women. Interventions developed based on the identified risk factors may prove effective in addressing the issue of malnutrition within this population.

## Introduction

The global population of older adults, aged 60 years and older, is considerably large. According to the World Health Organization, the global population of individuals aged 60 years and older was approximately 1 billion in 2019. It is projected to reach 1.4 billion by 2030 and further increase to 2.1 billion by 2050 [1]. Older adults in Saudi Arabia are expected to increase from 5.6% in 2017 to 23% by 2050 [2]. This rapid growth emphasizes the importance of the country to prepare and adapt to the changing demographics.

The process of aging involves a gradual decline in physiological functions, resulting in a decrease in the functional abilities of various organ systems and a reduced ability to cope with physical, cognitive, and mental stressors [3]. The impact of these changes can have a profound effect on the overall well-being of individuals. Additionally, as aging progresses, the prevalence of chronic diseases and multiple chronic conditions increases substantially, placing significant burdens on both individuals and the health care system. Older adults face greater risks of experiencing adverse health outcomes and complications, including malnutrition [4].

The prevalence of malnutrition among older adults is substantial and varies across different countries, ranging from an estimated 13% to 54% globally [5]. According to a recent study conducted in Nepal, the occurrence of malnutrition among a group of 320 older adults was found to be 11.6% [6]. The earlier study found that malnutrition was more common among women (15%) compared to men (9.3%). However, malnutrition is a complex condition influenced by various factors that can vary between countries and even different cities within the same country [3]. Therefore, this study specifically focuses on the western region of Saudi Arabia to examine the prevalence of malnutrition and its associated factors among older adults, taking into account the variations based on sex.

## Methods

### Participants

This study was conducted in the western region of Saudi Arabia (Makkah, Jeddah, Al-Taif, and Al-Madinah) as a community-based, nonrandomized, cross-sectional study, and was conducted between the periods of October 2022 and December 2022. To be included in the study, participants had to be older adults aged <60 years and living in the western region of Saudi Arabia. However, hospitalized older adults were excluded from the study. Out of 883, 623 were included; 2 were missing data, and 258 were out of the recruited age. As per the latest data from the General Authority for Statistics Report in the Kingdom of Saudi Arabia (KSA), there are approximately 400,000 older adults residing in the western region. The sample size was calculated using the Raosoft website. Based on specific parameters, including a 5% accepted margin of error, a 95% CI, and a 50% response distribution, the minimum required sample size for the study was determined to be 384 older adults.

### Study Design

A web-based questionnaire was submitted to eligible participants. Informed consent was meticulously drafted in simple Arabic language to ensure understanding, and it was obtained directly from the older adults or through their immediate family members, as outlined in the procedural instructions prior to questionnaire completion. Personal information was obtained by a closed questionnaire, including age, height (cm), weight (kg), BMI (kg/m^2^), nationality, city, social status, educational level, family income, living status, and health status. The Mini Nutritional Assessment (MNA) Short Form was used to determine the malnutrition status of older adults [7]. The MNA Short Form questionnaire includes 6 sections: food intake declined over the past 3 months due to loss of appetite, digestive problems, chewing or swallowing difficulties, weight loss during the last 3 months, mobility, experiencing psychological stress or acute disease in the past 3 months, neuropsychological problems, and BMI (kg/m^2^). Each answer to the previous questions is assigned a score, and the total value is the MNA score. Based on this score, we divided the sample into 2 categories: normal (MNA score ranging from 8 to 14), and malnourished (MNA score ≤7 points).

### Statistical Analysis

To analyze the data statistically, we applied IBM-SPSS statistics (version 23). We considered a *P* value less than .05 as statistically significant. For continuous variables, we represented them as the mean and SD. On the other hand, category variables were compared using a chi-squared test to examine their frequencies. The Mann-Whitney test was used to compare the mean ranks between the 2 groups when the distribution was not normal. To evaluate the risk factors associated with malnutrition, we conducted a multivariate binary logistic regression separately for men and women. This analysis allowed us to determine the odds ratio (OR) along with the corresponding 95% CI.

### Ethical Considerations

This study was approved by the Ethical Committee of Umm Al-Qura University (approval number AMSEC-6-954-2022), following the Declaration of Helsinki. All participants in the study volunteered and provided informed consent at the beginning. The data collected was made anonymous and did not include any identifiable information. As a token of appreciation, all participants who completed the questionnaire received a US $25 gift voucher.

## Results

The findings of this study are presented in Tables 1-3. Table 1 presents the sociodemographic characteristics of the participants in relation to malnutrition in both sexes. Tables 2 and 3 present the findings of logistic regression analysis, which was used to assess the probability of malnutrition occurrence based on the presence or absence of various risk factors in men and women, respectively. As shown in Table 1, of the total sample size (n=623), 36.5% were male and 55.5% were female. The majority of participants (95.3%) were of Saudi Arabian nationality. The prevalence of malnutrition was observed to be slightly higher among men (≈7%) compared to women (≈5%). Age was identified as a non-modifiable risk factor (a trait that cannot be altered or changed) that showed a positive association with malnutrition in older men. This suggests that as men get older, they are more likely to experience malnutrition (OR 1.263; 95% CI 1.086-1.468; *P*=.002; Table 2). However, no such association was observed among women (Table 3).

As opposed to the positive association detected between malnutrition and age, a negative correlation was observed between malnutrition and BMI in women. Specifically, the incidence of malnutrition was found to be more prevalent among women with a lower BMI compared to those with a higher BMI (mean BMI 25.5, SD 8.1 vs mean 29.4, SD 7; *P*=.04; Table 1). Logistic regression analysis further supported this finding, indicating that older women with a higher BMI had a 16% lower probability of experiencing malnutrition compared to those with a lower BMI (OR 0.843, 95% CI 0.747-0.952; *P*=.006), as depicted in Table 3.

As presented in Table 1, additional factors were found to be associated with the nutritional status of both male and female older adults. These factors include educational level, social status, and health status. Specifically, individuals with higher levels of education exhibited better health outcomes across both sexes. For instance, a significant proportion of older men (76.3%) who held bachelor's or postgraduate degrees were not affected by malnutrition, whereas only 35% experienced malnutrition (*P*=.001). Similarly, 62.9% of women, with bachelor's or postgraduate degrees did not experience malnutrition, while only 29.4% were malnourished (*P*=.02) (Table 1). Furthermore, malnutrition was less prevalent among financially independent individuals of both sexes. Notably, 93.8% of financially independent men were not malnourished, whereas only 6.2% of them experienced malnutrition (*P*<.001). Likewise, among financially independent women, 79% were not malnourished, while 21% of them exhibited malnutrition (*P*=.005).

Regarding social status, being married was associated with a more favorable nutritional status, with 90.7% of married men not affected by malnutrition. Additionally, the presence of a spouse and children in the household was linked to a significantly lower prevalence of malnutrition among older adults. Around 96% of men living with their wives and children were not affected by malnutrition, while only 3.9% experienced malnutrition (*P*=.006; Table 1). Conversely, being widowed was identified as a risk factor for malnutrition in older men. Interestingly, according to the logistic regression analysis, widowed men had an approximately 8-fold higher likelihood of experiencing malnutrition compared to their married counterparts (95% CI 1.002-70.258; *P*=.049), as depicted in Table 2. However, this association was not evident among women (Table 3).

In terms of health status, the prevalence of malnutrition was observed to be high among men with chronic health conditions. Compared to healthy older men, malnutrition was notably more prevalent among individuals diagnosed with diabetes (15% vs 85%, *P*=.006), hypertension (20% vs 80%, *P*=.007), and heart disease (40% vs 60%, *P*=.001; Table 1). An additional noteworthy factor linked to the likelihood of developing malnutrition in older men was the presence of dental problems. Specifically, older men with dental problems exhibited a significantly higher probability of experiencing malnutrition, approximately 9-fold higher than those without dental problems (95% CI 1.863-47.514; *P*=.007; Table 2). As for women, disability was found to be strongly associated with malnutrition. The probability of experiencing malnutrition in older women with a disability was approximately 18-fold higher compared to their counterparts without a disability (95% CI 2.605-125.626; *P*=.003; Table 3).

**Table 1:** Relationship between malnutrition and participants’ characteristics based on sex.

Variable		Men (n=277)			*P* value			Women (n=346)					*P* value
		Malnutrition						Malnutrition					
		Yes (n=20)	No (n=257)				Yes (n=17)			No (n=329)			
Age (years), mean (SD)		73.1 (7.6)	65.3 (4.9)	*<.001* ^a^			66.6 (5.7)			64.3 (5.3)		*.03*	
Height (cm), mean (SD)		178.9 (5.9)	171.7 (7)	.08			157.4 (7.5)			159.3 (7)		.37	
Weight (kg), mean (SD)		84.8 (26)	84.1 (15.9)	.57			62.8 (18.7)			74.4 (17.7)		.15	
BMI (kg/m^2^), mean (SD)		26.5 (8.1)	28.4 (5.4)	.05			25.5 (8.1)			29.4 (7)		*.04*	
MNA^b^ score, mean (SD)		5.2 (1.5)	12.5 (1.6)	*<.001*			6 (1.1)			12.1 (1.8)		*<.001*	
**Nationality, n (%)**						.30							.51
	Saudi	18 (90)	244 (94.9)				16 (94.1)			316 (96)			
	Non-Saudi	2 (10)	13 (5.1)				1 (5.9)			13 (4)			
**Residence, n (%)**						.19							.34
	Makkah	11 (55)	127 (49.4)				12 (70.6)			220 (66.9)			
	Jeddah	5 (25)	99 (38.5)				3 (17.6)			83 (25.1)			
	Al-Taif	3 (15)	12 (4.7)				0 (0)			13 (4)			
	Al-Madinah	1 (5)	19 (7.4)				2 (11.8)			13 (4)			
**Social status, n (%)**						*<.001*							*.007*
	Married	9 (45)	233 (90.7)				4 (23.5)			202 (61.4)			
	Single or divorced	2 (10)	11 (4.3)				6 (35.3)			51 (15.5)			
	Widowed	9 (45)	13 (5)				7 (41.2)			76 (23.1)			
**Education level, n (%)**						*.001*							*.009*
	Illiterate or elementary	5 (25)	14 (5.4)				5 (29.4)			56 (17)			
	Middle school	2 (10)	8 (3.1)				4 (23.5)			19 (5.8)			
	High school	6 (30)	39 (15.2)				3 (17.6)			47 (14.3)			
	Bachelor’s degree	4 (20)	132 (51.4)				3 (17.6)			178 (54.1)			
	Postgraduate degree	3 (15)	64 (24.9)				2 (11.8)			29 (8.8)			
**Income (SAR^c^), n (%)**						*.04*							*.02*
	<5000	6 (30)	24 (9.3)				11 (64.7)			99 (30.1)			
	5001-10,000	4 (20)	55 (21.4)				2 (11.8)			81 (24.6)			
	10,001-20,000	5 (25)	99 (38.5)				2 (11.8)			117 (35.6)			
	>20,000	5 (25)	79 (30.7)				2 (11.8)			32 (9.7)			
**Describe your financial status, n (%)**						*<.001*							*.005*
	Dependent	8 (40)	16 (6.2)				9 (52.9)			69 (21)			
	Independent	12 (60)	241 (93.8)				8 (47.1)			260 (79)			
**Living status, n (%)**						*.01*							*.008*
	Alone	4 (20)	10 (3.9)				8 (47.1)			60 (18.2)			
	With wife, husband, or children	16 (80)	247 (96.1)				9 (52.9)			269 (81.8)			
**Diabetes, n (%)**						*.006*							.10
	Yes	17 (85)	140 (54.5)				10 (58.8)			131 (39.8)			
	No	3 (15)	117 (45.5)				7 (41.2)			198 (60.2)			
**Hypertension, n (%)**						*.007*							.47
	Yes	16 (80)	127 (49.4)				9 (52.9)			161 (48.9)			
	No	4 (20)	130 (50.6)				8 (47.1)			168 (51.1)			
**Heart disease, n (%)**						*.001*							.18
	Yes	12 (60)	62 (24.1)				4 (23.5)			42 (12.8)			
	No	8 (40)	195 (75.9)				13 (76.5)			287 (87.2)			
**Disability, n (%)**						*<.001*							*.001*
	Yes	12 (60)	13 (5.1)				4 (23.5)			7 (2.1)			
	No	8 (40)	244 (94.9)				13 (76.5)			322 (97.9)			
**Dental problem, n (%)**						*<.001*							.19
	Yes	18 (90)	112 (43.6)				12 (70.6)			186 (56.5)			
	No	2 (10)	145 (56.4)				5 (29.4)			143 (43.5)			
**Smoking, n (%)**						.12							.40
	Yes	6 (30)	72 (28)				1 (5.9)			34 (10.3)			
	Ex-smoker	5 (25)	118 (45.9)				16 (94.1)			271 (82.4)			
	No	9 (45)	67 (26.1)				0 (0)			24 (7.3)			

^a^The *P* values presented in italics are considered statistically significant at *P*<.05.

^b^MNA: Mini Nutritional Assessment.

^c^SAR: Saudi Riyals (SAR 1=US $0.27).

**Table 2:** Potentially significant predictors related to malnutrition for men.

Variable		Odds ratio	95% CI	*P* value
Age		1.263	1.086-1.468	*.002* ^a^
**Social status**				
	Married	1^b^	—^c^	—
	Single or divorced	1.603	0.063-40.822	.78
	Widowed	8.392	1.002-70.258	*.049*
**Education level**				
	Illiterate or elementary	3.931	0.176-87.737	.39
	Middle school	1.774	0.047-66.967	.76
	High school	7.023	0.368-134.086	.20
	Bachelor’s degree	0.804	0.073-8.874	.86
	Postgraduate degree	1	—	—
**Income (SAR^d^)**				
	<5000	3.893	0.182-83.176	.38
	5001-10,000	0.287	0.012-6.945	.44
	10,001-20,000	1.443	0.128-16.266	.77
	>20,000	1	—	—
**Describe your financial status**				
	Dependent	0.462	0.052-4.12	.49
	Independent	1	—	—
**Living status**				
	Alone	4.299	0.258-71.566	.31
	With wife, husband, or children	1	—	—
**Diabetes**				
	Yes	0.544	0.07-4.233	.56
	No	1	—	—
**Hypertension**				
	Yes	1.84	0.304-11.128	.51
	No	1	—	—
**Heart disease**				
	Yes	3.048	0.52-17.851	.22
	No	1	—	—
**Disability**				
	Yes	6.666	0.741-59.986	.09
	No	1	—	—
**Dental problem**				
	Yes	9.408	1.863-47.514	*.007*
	No	1	—	—

^a^Italic values are considered statistically significant.

^b^Reference values.

^c^Not applicable.

^d^SAR: Saudi Riyals (SAR 1=US $0.27).

**Table 3:** Potentially significant predictors related to malnutrition for women.

Variable		Odds ratio	95% CI	*P* value
Age		1.017	0.925-1.118	.73
BMI		0.843	0.747-0.952	*.006* ^a^
**Social status**				
	Married	1^b^	—^c^	—
	Single or divorced	3.099	0.456-21.082	.25
	Widowed	2.653	0.492-14.303	.26
**Education level**				
	Illiterate or elementary	0.703	0.036-13.776	.82
	Middle school	1.371	0.071-26.494	.84
	High school	0.479	0.027-8.492	.62
	Bachelor’s degree	0.457	0.047-4.431	.50
	Postgraduate degree	1	—	—
**Income (SAR^d^)**				
	<5000	1.284	0.114-14.451	.84
	5001-10,000	0.357	0.024-5.214	.45
	10,001-20,000	0.37	0.039-3.538	.39
	>20,000	1	—	—
**Describe your financial status**				
	Dependent	1.749	0.468-6.546	.41
	Independent	1	—	—
**Living status**				
	Alone	1.76	0.422-7.336	.44
	With wife, husband, or children	1	—	—
**Disability**				
	Yes	18.089	2.605-125.626	*.003*
	No	1	—	—

^a^Italic values are considered statistically significant.

^b^Reference value.

^c^Not applicable.

^d^SAR: Saudi Riyals (SAR 1=US $0.27).

## Discussion

### Principal Findings

This study explored the prevalence of malnutrition among older men and women in the Saudi Arabian community in the western province, with an emphasis on its associated risk factors. The study revealed that the occurrence of malnutrition was approximately 7% among men and 5% among women. This is comparable to the findings of studies conducted in the region. For instance, a study conducted in Jeddah city reported that the prevalence of malnutrition among older adults was 5.3% [8]. Another study conducted in Al Madinah Al Munawarah reported a lower prevalence of malnutrition among older adults, accounting for 3.5% [9]. One possible reason for this difference in the prevalence of geriatric malnutrition could be attributed to the different support and health programs provided in each district.

However, a study involving participants from Makkah and Jeddah cities found a higher prevalence of malnutrition among older adults, reaching approximately 13% [10]. This elevated prevalence could be linked to the study's recruitment strategy, which involved recruiting individuals from public places as well as health care clinics. This suggests that the study cohort may have had preexisting health conditions or chronic illnesses that could impact their nutritional status. This is consistent with a further study conducted in Jeddah city, which included hospitalized patients and reported a higher prevalence of geriatric malnutrition, reaching 27% [11].

The association between malnutrition and chronic disease is well established in the literature [12]. This is also confirmed by the findings of this study, which found that the prevalence of malnutrition was higher among older men with chronic health conditions (ie, diabetes, hypertension, and heart disease), which could be attributed to several factors. For instance, symptoms associated with chronic diseases, such as fatigue, pain, or shortness of breath, can impact appetite and food intake, leading to malnutrition [13]. Furthermore, medications used to manage chronic diseases can have side effects that reduce appetite, alter taste perception, or impair nutrient absorption, leading to malnutrition [14]. Therefore, regular monitoring of the old individual's nutritional status as well as any signs of malnutrition is recommended in order to identify and address any deficiencies or imbalances early on.

The significant association between chronic disease and malnutrition observed in men, but not in women, could be partially explained by the higher prevalence of chronic conditions in the male subsample compared to the female subsample. Specifically, the prevalence of diabetes was 85% in men versus 58.8% in women, the prevalence of hypertension was 80% in men versus 52.9% in women, and the prevalence of heart disease was 60% in men versus 23.5% in women. This disparity in chronic disease burden between sexes may have contributed to the stronger link between chronic conditions and malnutrition risk observed in the male participants. The higher underlying chronic disease prevalence in the male group could make them more susceptible to the detrimental effects of chronic illness on nutritional status, leading to the significant association found in the regression analysis. However, further investigation into the complex interplay of sex, chronic disease burden, and nutritional status in this population would be valuable to elucidate the factors contributing to the divergent findings.

Not only were chronic health conditions associated with the prevalence of malnutrition in the participated sample, but disability was also a strong predictor, especially among women. In this study, women with disabilities were 18 times more likely to experience malnutrition compared to healthy individuals. This is consistent with a study conducted in the KSA, which reported that physically dependent older adults had higher scores of malnutrition compared to their independent counterparts [15]. This could be explained by the fact that disability adversely affects the potential to access and consume nutritious food, leading to poor nutritional intake [16]. In contrast to the adverse impact of disability on nutritional status, mobility in older patients was associated with 90% lower cases of malnutrition compared to being bed-bound, as shown by a recent study conducted in the KSA [11]. This is probably because older adults with better mobility are more likely to be independent in performing their daily activities, including shopping, cooking, and eating. This independence allows them to have greater autonomy in their food choices and meal preparation, leading to a higher likelihood of consuming a balanced and nutritious diet [17].

Contrary to the significant negative association observed between disability and nutritional status among women, this study did not detect a comparable adverse effect of physical impairment on the nutritional profile of men. This difference may be partly explained by the slightly higher prevalence of disability among women (46.7%) compared to men (41.5%) in the KSA, based on openly available data from 2015 to 2020 [18]. Another potential explanation for the significant impact of disability on women, compared with the absence of such an association in men, may lie in the social context. For instance, physical impairment might be linked with social isolation and depression [19], which could diminish appetite and subsequently lower nutritional intake, resulting in malnutrition [20]. According to the World Health Organization, rates of depression are higher among women (6%) compared to men (4%) [21]. This discrepancy may elucidate the observed sex differences in the relationship between disability and nutritional status.

According to the findings of this study, dental problems were also identified as a risk factor for geriatric malnutrition. The likelihood of experiencing malnutrition in older men was approximately 9 times higher in participants experiencing dental problems compared to their counterparts who had good dental health. This is in line with the findings of a recent study conducted in Riyadh (central region, KSA), which reported that malnutrition was greater in patients with higher scores of oral health problems compared to those with lower scores [22]. This is expected, as dental and oral problems in older adults could be associated with malnutrition due to difficulties in chewing, pain, and discomfort while eating, leading to poor nutrient intake.

In this study, we investigated the relationship between malnutrition among older adults and BMI as a continuous variable, rather than focusing on a specific weight status category such as underweight or overweight. It is important to note that obesity is prevalent in the KSA [23]; however, there are limited data regarding the associations between weight status and malnutrition among older adults in the country. While low BMI is often used to identify the presence of malnutrition [24], we sought to explore the associations between an individual's BMI, across its entire range, and the likelihood of being malnourished. The analysis took into account the complex interplay of factors that can contribute to malnutrition risk. This allowed us to assess the independent effect of BMI, treated as a continuous measure, on malnutrition, while controlling for other potential confounding variables. It was found that a higher BMI is considered a protective factor against malnutrition in the participated older women. This is in alignment with the findings of several studies conducted in Kuwait [25], Lebanon [26], and Iran [27]. The reduced risk of malnutrition among older adults with a higher BMI could be explained by the fact that a higher BMI implies larger energy reserves and nutrient stores, which can help buffer against periods of reduced food intake or increased nutrient requirements that may occur in situations of illness or decreased appetite [28]. However, a recent review highlighted the absence of comprehensive research on anthropometric cutoffs for older adults in Saudi Arabia, which hampers the development of evidence-based guidelines. Thus, further studies are needed to establish anthropometric cutoffs that align with the Saudi Vision 2030 [29]. This is important for enabling health care professionals to accurately assess and manage the nutritional needs of older adults.

According to the study findings, being married and living with family emerged as potential protective factors against malnutrition in older men. This is expected and could be attributed to the positive influence of social interaction and companionship during mealtimes, which could enhance appetite and enjoyment of food. In contrast, being widowed in men was associated with 8 times greater likelihood of experiencing malnutrition compared to being married. This is in line with the findings of a systematic review and meta-analysis of 40 observational studies, which confirmed that social status is a predictor of geriatric malnutrition [30]. The adverse influence of spouse loss on malnutrition in older adults might be due to a lack of emotional support and companionship, which can contribute to a decline in appetite and skipping meals, leading to poor nutritional status [31,32].

It is worth mentioning that the absent probability of experiencing malnutrition in women compared to men, in this study, might be explained by cultural norms and sex roles. In many societies, traditional sex roles have assigned women the primary responsibility for food preparation, cooking skills, and attention to the nutritional value of food. This has resulted in women having a greater understanding of nutrition and being less prone to malnutrition compared to men who may rely on their wives for meal preparation [33].

The preceding sections discussed the findings of this study in terms of the prevalence of geriatric malnutrition in the KSA and its associated risk factors. This section will briefly discuss the potential solutions to address this pressing issue. It is recommended to consider comprehensive interventions that encompass a range of strategies aimed at mitigating malnutrition among older adults. These strategies may include social support, dietary modifications, nutritional supplementation, meal delivery programs, and nutrition education or counseling [34-36]. Moreover, it is important to consider cultural factors during the development of the intervention, as they play a crucial role in shaping dietary habits, attitudes towards food, and health-seeking behaviors in older adults [37]. By designing an intervention that aligns with the cultural norms, values, and preferences of the target population, the effectiveness and acceptability of the intervention are likely to be enhanced [38]. Therefore, it is recommended to develop culturally tailored interventions that tackle barriers including traditional food practices, religious beliefs, and social norms, as they are likely to have the potential to enhance nutrition outcomes.

Furthermore, it is recommended to leverage the advantages offered by modern technology and widespread internet usage, along with the proliferation of handheld devices. These factors could facilitate intervention delivery to older adults. This is supported by the findings of a systematic review of 70 studies, which indicated that eHealth interventions using mobile apps could be deemed effective in enhancing health outcomes in older adults [39]. Nevertheless, that review underscored heterogeneity in terms of intervention duration, targeted health outcomes, and populations. Therefore, additional research endeavors are warranted in this domain to establish robust evidence concerning the optimal approaches for addressing geriatric malnutrition.

### Limitations

It is important to note that this study has a cross-sectional design, which limits its scope. Moreover, older adults were only recruited from certain regions in the KSA, so the findings of this study cannot be generalized to the entire older adults in the KSA. Another limitation of the study involved the self-reported nature of the data. The researchers relied on self-reported information from the participants, which may be subject to recall bias or social desirability bias. This could potentially affect the accuracy of the data collected, particularly regarding factors such as weight loss, food intake, and health status. However, a key strength of the study is that, to the best of our knowledge, it is the first to investigate the sex-based differences in the prevalence and associated risk factors of malnutrition among older adults in the KSA. This represents an important contribution to the limited existing literature on this topic in the KSA.

### Conclusions

The findings of this study indicate that malnutrition affects both older men and women in the western region of Saudi Arabia. Importantly, the study identified various risk factors associated with malnutrition in this population. These findings hold significance for informing the development of targeted interventions aimed at addressing malnutrition among older adults. By identifying the specific risk factors, health care providers and policy makers can tailor strategies and interventions to effectively meet the distinct needs and challenges faced by both sexes. Furthermore, understanding the impact of malnutrition on older men and women allows for the consideration of sex-specific factors in the planning and implementation of interventions. This recognition enables the design of interventions that account for the unique circumstances, social dynamics, and cultural norms that may influence nutritional health differently for men and women. In addition, future research is recommended to evaluate the efficacy of digital interventions in mitigating malnutrition within the Saudi Arabian population, considering the potential benefits of technology-based approaches to addressing this issue.

## Abbreviations

KSA: Kingdom of Saudi Arabia
MNA: Mini Nutritional Assessment
OR: odds ratio
